# Pediatric Versus Adult Nasopharyngeal Cancer in Diffusion-Weighted Magnetic Resonance Imaging

**DOI:** 10.3390/cancers17132237

**Published:** 2025-07-03

**Authors:** Emil Crasnean, Ruben Emanuel Nechifor, Liviu Fodor, Oana Almășan, Nico Sollmann, Alina Ban, Raluca Roman, Ileana Mitre, Simion Bran, Florin Onișor, Cristian Dinu, Mihaela Băciuț, Mihaela Hedeșiu

**Affiliations:** 1 Department of Maxillofacial Surgery and Implantology, Faculty of Dentistry, “Iuliu Hatieganu” University of Medicine and Pharmacy, 400012 Cluj-Napoca, Romania; 2 Department of Clinical Psychology and Psychotherapy, International Institute for the Advanced Studies of Psychotherapy and Applied Mental Health, Babeș-Bolyai University, 400489 Cluj-Napoca, Romania; ruben.nechifor@ubbcluj.ro (R.E.N.);; 3 Institute for Research, Development and Innovation in Applied Natural Science, Babes-Bolyai University, 30 Fântanele, 400327 Cluj-Napoca, Romania; 4 Department of Prosthetic Dentistry and Dental Materials, “Iuliu Hațieganu” University of Medicine and Pharmacy, 32 Clinicilor Street, 400006 Cluj-Napoca, Romania; 5 Department of Diagnostic and Interventional Radiology, Ulm University Hospital, 89081 Ulm, Germany; 6 Department of Diagnostic and Interventional Neuroradiology, School of Medicine, Klinikum Rechts der Isar, Technical University of Munich, 81675 Munich, Germany; 7 TUM-Neuroimaging Center, Klinikum Rechts der Isar, Technical University of Munich, 81675 Munich, Germany

**Keywords:** magnetic resonance imaging, nasopharyngeal carcinoma, diffusion-weighted imaging, pediatric, adults

## Abstract

**Background:** This study aimed at evaluating apparent diffusion coefficient (ADC) values of nasopharyngeal carcinoma (NPC) in the pre-treatment stages of NPC for establishing comparative quantitative parameters between children and adolescents compared to adults. **Methods:** A retrospective multicentric imaging study was conducted in three medical centers by collecting patient data over a 5-year timeframe. Patients were included in the study based on the following criteria: histopathologically proven carcinoma of the nasopharynx with all available medical records. The total sample included 20 patients (6 pediatric patients and 14 adults). A quantitative analysis of the ADC maps was performed. Two radiologists manually drew the region of interest (ROI) on ADC maps using the whole tumor on all magnetic resonance imaging (MRI) slices. The mean ADC was extracted for each patient and each radiologist’s evaluation. Differences in ADC values between pediatric and adult patients were evaluated using an independent samples *t*-test, with normality and variance assumptions tested via the Shapiro–Wilk and Levene’s tests, respectively. *p*-values less than 0.05 were considered statistically significant. **Results:** The mean ADC values extracted from the initial pre-treatment diffusion-weighted imaging (DWI) data from magnetic resonance imaging (MRI) in children were 712.22 × 10^−6^ mm^2^/s, compared to adults in whom the mean ADC values were 877.34 × 10^−6^ mm^2^/s. We found a statistically significant difference between the mean ADC values of pediatric patients and adult patients, t (17.44) = −3.15, *p* = 0.006, with the mean ADC values of pediatric patients (M = 712.22, standard deviation [SD] = 57.03) being lower, on average, than the mean ADC values of adult patients (M = 877.34, SD = 175.25). **Conclusions:** Our results showed significantly lower ADC values in pediatric patients than in adults, independent of tumor T-stage. Additionally, early-stage tumors, particularly in children, tended to exhibit even lower ADC values, suggesting potential biological distinctions across age groups.

## Simple Summary

The pre-treatment apparent diffusion coefficient (ADC) of the primary lesion may reflect the intrinsic biological characteristics of a tumor and provide additional valuable information regarding T classification. As a non-invasive method, diffusion-weighted imaging (DWI) from magnetic resonance imaging (MRI) can support the characterization of nasopharyngeal carcinoma (NPC) in both adult and pediatric patients. This study aimed to evaluate the pre-treatment ADC values of NPC to establish comparative quantitative parameters between pediatric and adult cohorts. We found a statistically significant difference in mean ADC values, with pediatric patients exhibiting lower values than adults. Importantly, this difference was independent of tumor T-stage, and early-stage tumors, particularly among pediatric cases, tended to have even lower ADC values, especially in pediatric patients.

## 1. Introduction

Head and neck (HNC) cancers are rare in childhood and represent 12% of all pediatric malignancies [1]. About 1 in 10 childhood cancer cases is associated with the HNC region and the most common types are Hodgkin and non-Hodgkin lymphomas (27%), neural tumors, thyroid cancer (21%), rhabdomyosarcomas (RMS) (23%), nasopharyngeal carcinoma (NPC), skeletal malignancies, salivary gland malignancies, and neuroblastomas [2,3]. Nasopharyngeal malignancies are particularly rare, with an overall incidence of less than 1 per 100,000 worldwide with considerable geographic and racial variations (i.e., southern parts of China, Southeast Asia, Alaska, and the Mediterranean Basin) [4]. The most frequent pediatric NPC entity is the undifferentiated type of carcinoma [5]. On the other hand, adult cancers that arise from the nasopharynx are usually squamous cell carcinomas (SCCs) that behave differently than the other HNC types through insidious onset or may have a prolonged history of the disease before the diagnosis [6].

The World Health Organization (WHO) classification system for NPC characterizes the tumors based on the following histopathologic subtypes: keratinizing SCC (type I), non-keratinizing differentiated or undifferentiated carcinoma (type II), and basaloid SCC (type III), a rare histopathologic form [7]. In the pediatric population, the first peak of incidence of NPC is between 10 and 20 years of age, with a median age of 13 years at the time of diagnosis [5]. Epstein–Barr virus (EBV), environmental influence, and genetics are associated with this type of carcinoma, and also increased antibody titers of immunoglobulin G (IgG) and immunoglobulin A (IgA) are seen in children with undifferentiated carcinoma [8].

Both in the pediatric and adult population, when the nasopharynx is involved, the symptoms typically include nasal, neurological, and auditory problems. Nasal obstruction and epistaxis, otalgia, hearing loss, cranial nerve neuropathies, neck lumps, neck mass, and headaches are the most frequently encountered symptoms [9]. A delayed diagnosis can be found in children compared to adults because of these symptoms usually being of insidious onset.

Treatment of NPC in childhood has generally followed guidelines established for adults, with high-dose radiotherapy (RT) to the nasopharynx and involved neck lymph nodes [10]. However, childhood NPC is distinguished from adult NPC by its close association with EBV, predominance of type III histology, and high incidence of advanced-stage disease, in addition to the considerable morbidity of high-dose RT in childhood [10]. Extensive surgical resection, such as the maxillary swing procedure, does not apply to children due to skeletal immaturity, surgical-related morbidity, and concerns about incomplete resection [11]. This has led to an increase in trials of adjuvant, neoadjuvant, and concomitant chemotherapy [12,13]. Due to multimodal treatment approaches that combine chemotherapy and RT, the overall prognosis for pediatric NPC has significantly improved, with over 90% of patients achieving overall survival (OS) and progression-free survival (PFS) at 5 years, even in cases of primary advanced locoregional disease [13].

Magnetic resonance imaging (MRI), computed tomography (CT), and 18F-fluorodeoxyglucose (18F-FDG) positron emission tomography CT (PET/CT) are among the recommended imaging modalities for NPC [14,15,16,17]. Specifically, MRI has the advantages of high-quality tissue contrast, superior evaluation of primary tumor extension (parapharyngeal or oropharyngeal extension and ethmoid sinus involvement), and evaluation of retropharyngeal lymph node metastases [18]. Meanwhile, CT could be used in detecting bone changes such as erosion [19]. Moreover, PET/CT is used as the preferable conventional work-up method because of its higher sensitivity as it has advantages in the diagnosis of distant metastasis or in small cervical lymph node metastasis, as well as in the diagnosis of recurrences [20].

Diffusion-weighted imaging (DWI) from MRI is a sequence based on measuring the random Brownian motion of water molecules within tissues [21,22]. The apparent diffusion coefficient (ADC) reflects the diffusion speed of water molecules [23]. Tumors often show impaired cell membranes that can cause water restriction [17]. In this regard, an effective treatment in NPC can lead to a decrease in tumor cellularity so it can produce a higher ADC value [24]. Furthermore, ADC values are inversely proportional to tumor cellularity [25]. Hence, DWI-MRI has offered high diagnostic potential by its capacity to review tissue cellularity, the integrity of cell membranes, and the viscosity of fluids, thus enabling tissue characterization [26]. As a non-invasive method, DWI-MRI could facilitate characterization of NPC, both in adult patients as well as pediatric cohorts. Given that NPC presents differently across age groups, particularly with distinct biological behavior, clinical manifestations, and treatment responses in children, adolescents, and adults, it is critical to investigate how ADC values vary in these populations. Understanding the age-specific differences in ADC could enhance diagnostic accuracy and guide personalized treatment strategies.

While there is a clear need for age-specific diagnostic and treatment approaches in NPC, no previous studies have directly investigated potential differences in ADC values between pediatric and adult patients with NPC. Our study intends to address this gap by evaluating pre-treatment ADC values in nasopharyngeal carcinoma (NPC) by establishing comparative quantitative parameters between pediatric (children and adolescents) and adult patients. Identifying such differences could have important implications for improving the role of DWI in NPC characterization and guiding age-tailored treatment strategies.

## 2. Materials and Methods

### 2.1. Patients

The study was approved by the institutional review boards of the participating centers (Cluj Napoca approval number DEP 45/4532/2022; Ulm approval number 392/22). The requirement for written informed consent was waived due to the retrospective study design.

A retrospective multicentric imaging study was conducted in three medical centers (Oncology Institute “Prof. Dr. Ion Chiricuţă” Cluj-Napoca, Romania, Ulm University Hospital, Germany, and Emergency Hospital, Cluj-Napoca, Romania), by retrospectively collecting patient data over a 5-year timeframe (between January 2019 and January 2024). Patients were included in the study based on the following criteria: histopathologically proven carcinoma of the nasopharynx with all available medical records (pre-treatment DWI-MRI and histopathological exams) and age younger than 18 years (for the pediatric population) or over 18 years (for the adult population). The exclusion criteria were as follows: histopathology diagnosis other than carcinoma, cancer affecting other HNC regions than the nasopharynx (oral cavity, oropharynx, hypopharynx, nasal cavity, larynx, paranasal sinuses), treatment before the initial MRI, no DWI-MRI available in the pre-treatment stage, and artifacts on DWI-MRI. The total sample included 20 patients (6 pediatric patients and 14 adults). All participants in both the pediatric and adult groups were diagnosed with the same histological subtype, differentiated or undifferentiated non-keratinizing carcinoma, consistent with the WHO 2017 classification.

### 2.2. MRI Scanning

Structural and DWI-MRI data were obtained on GE Signa Explorer 1.5T (GE Healthcare, Milwaukee, WI, USA), Siemens Avanto or Area 1.5T (Siemens Healthineers, Erlangen, Germany), or Siemens Skyra, Spectra, or Vida Fit 3T MRI systems (Siemens Healthineers, Erlangen, Germany). The MRI data contained a variety of imaging sequences (the study was conducted across three research centers or universities and given the rarity of this pathology, data collection from multiple locations was necessary to recruit enough patients and increase statistical power). However, scans that were considered appropriate for imaging analysis included at least a T1-weighted sequence, a T2-weighted sequence, and DWI-MRI. Furthermore, the scans with movement artifacts that were of inadequate diagnostic quality were excluded. The ADC was computed from the DWI-MRI data, acquired with an echo time/repetition time = 62 to 102 ms/>5000 ms, flip angle of 90°, matrix size = 128 × 128 to 176 × 176, slice thickness = 2 to 6 mm, using multiple b-values of 0–80 and 800–1000 s/mm^2^ in 3 orthogonal directions.

### 2.3. Evaluation of Imaging Data

The DWI-MRI data were analyzed using Analysis of Functional NeuroImages (AFNI) software (version 23.11.10) [27]. A quantitative analysis of the ADC maps was performed by two specialist radiologists (M.H. with 25 years of experience, and R.R. with 20 years of experience), blinded to clinical data. Each of the imaging examiners analyzed the cases individually. The region of interest (ROI) was manually drawn on the axial view of the ADC maps by these two radiologists using an electronic cursor covering the whole tumor on all MRI slices. Structural images, including T2-weighted and contrast-enhanced T1-weighted sequences, were used to accurately localize tumor boundaries and identify non-viable regions. Cystic areas, visually hypointense on contrast-enhanced images and hyperintense on T2-weighted scans (consistent with necrosis), as well as any visible blood vessels, were excluded to avoid artificially high ADC values that may not reflect tumor cellularity. Apparent diffusion coefficient (ADC) values were extracted exclusively from the primary tumor region. Nodal metastases, when present, were not included in the ROI delineation to ensure consistency across subjects and to focus the analysis on the primary lesion’s diffusion characteristics.

**Figure 1:**
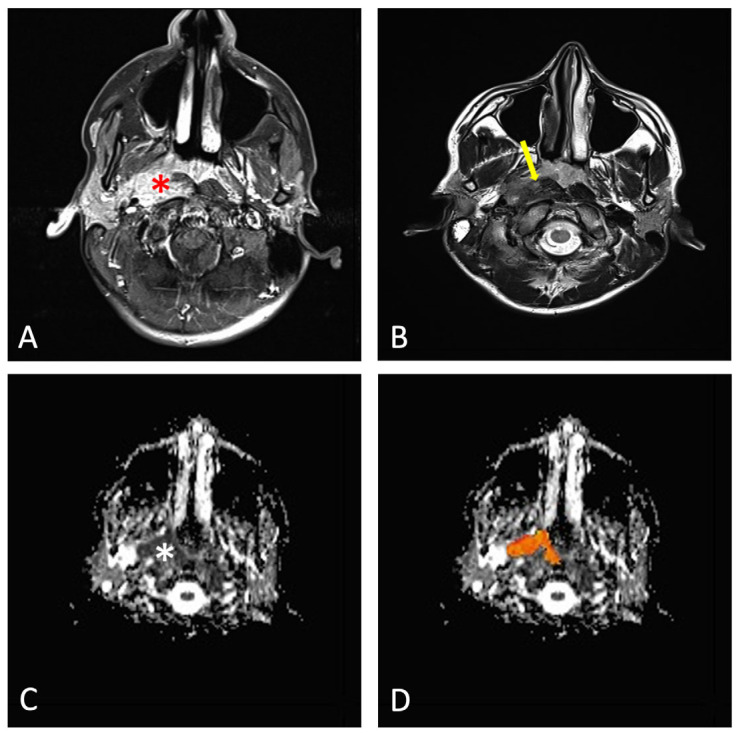
A 13-year-old child with NPC: (**A**) post-contrast axial T1-weighted imaging showing a large nasopharyngeal mass (red asterisk); (**B**) axial T2-weighted imaging demonstrating the involvement of the masticatory space (yellow arrow); (**C**) ADC map showing low signal intensity of the nasopharyngeal mass (white asterisk); (**D**) ROI was defined by covering as much tumor tissue as possible (shown on the slice with the maximum tumor diameter, orange area).

**Figure 2:**
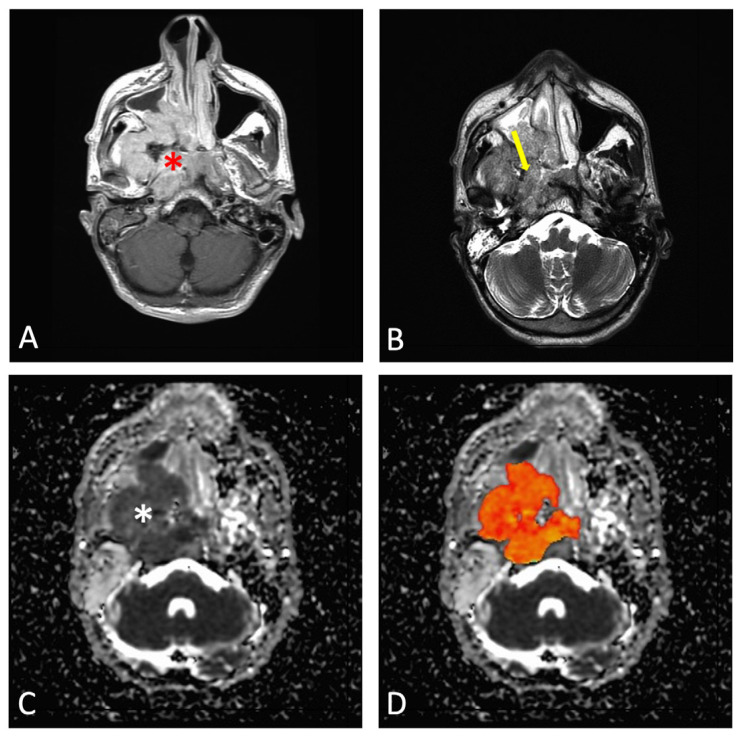
A 43-year-old male with NPC: (**A**) post-contrast axial T1-weighted imaging showing a large nasopharyngeal mass (red asterisk); (**B**) axial T2-weighted imaging demonstrating the involvement of the masticatory space (yellow arrow); (**C**) ADC map showing low signal intensity of the nasopharyngeal mass (white asterisk); (**D**) ROI was defined by covering as much tumor tissue as possible (shown on the slice with the maximum tumor diameter, orange area).

After the nasopharyngeal tumors were segmented, the mean ADC was extracted for each patient and each radiologist’s evaluation (Figure 1 and Figure 2).

### 2.4. Statistical Analysis

All statistical analyses were performed using the IBM SPSS (version 26) and JASP (version 0.19) statistical software [28,29].

**Figure 3:**
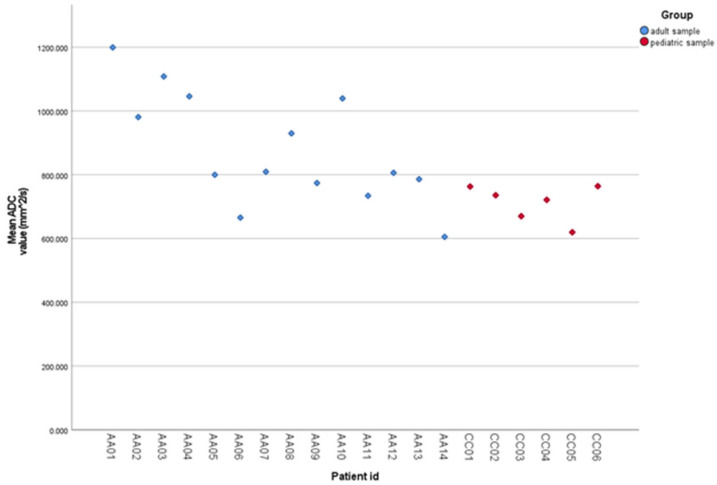
Scatter plot of individual ADC values stratified by sample type.

There was little variation between the mean ADC values that were derived from data independently evaluated by the two radiologists for the pediatric or adult patients (Figure 3), and we obtained a very high degree of inter-rater agreement, with an ICC of 0.98 (95% CI: 0.96 to 0.99).

We examined the data for severe outliers and missing values using descriptive statistics. We assessed any variations in mean ADC values between pediatric and adult patients for the main comparison. A new ADC value was computed for each case as an arithmetic mean of the two radiologists’ evaluations. An independent samples *t*-test was conducted to compare these mean ADC values between pediatric and adult patients, with Cohen’s d used as an indicator of the effect size. To check the assumptions that are necessary for performing the adequate *t*-test for the difference between ADC values of pediatric patients and adult patients (using the arithmetic mean between the ADC values of both radiologists), we performed the Shapiro–Wilk test of data distribution normality and Levene’s test for equality of variances. A *p*-value < 0.05 was considered statistically significant.

To account for the potential confounding effect of tumor T-stage, we also performed a two-way analysis of variance (ANOVA) with age group (pediatric vs. adult) and T-stage group (early vs. advanced) as fixed factors, including their interaction. The results indicated that age group had a statistically significant main effect on ADC values (F = 5.03, *p* = 0.040), while T-stage did not reach statistical significance (F = 2.89, *p* = 0.109), and no significant interaction was observed (F = 0.74, *p* = 0.403). These results suggest that the observed differences in ADC between pediatric and adult patients are not due to differences in tumor stage (Table 1).

## 3. Results

The total sample included 20 patients (6 children and 14 adults) who had undergone DWI-MRI in the pre-treatment stage for NPC. The median age of the children was 16 years (with an age range from 12 to 17 years), and the median age of the adults was 58 years (with an age range from 34 to 70 years). Demographic and clinical characteristics, including age distribution, gender, pathologic type, and staging (T, N, and overall stage), are summarized in Table 2.

The mean ADC extracted from the initial pre-treatment DWI-MRI in children was 712.22 × 10^−6^ mm^2^/s, compared to adults in whom the mean ADC was 877.34 × 10^−6^ mm^2^/s. The Shapiro–Wilk test indicated that the assumption of data distribution normality was met for both pediatric (W = 0.89, *p* = 0.316) and adult samples (W = 0.95, *p* = 0.617). Levene’s test was statistically significant (F (1, 18) = 8.53, *p* = 0.009), indicating that the equality of variances assumption was not met. For this reason, we employed the Welch *t*-test, rather than Student’s *t*-test, to evaluate the difference between the mean ADC values of pediatric patients and adult patients.

**Figure 4:**
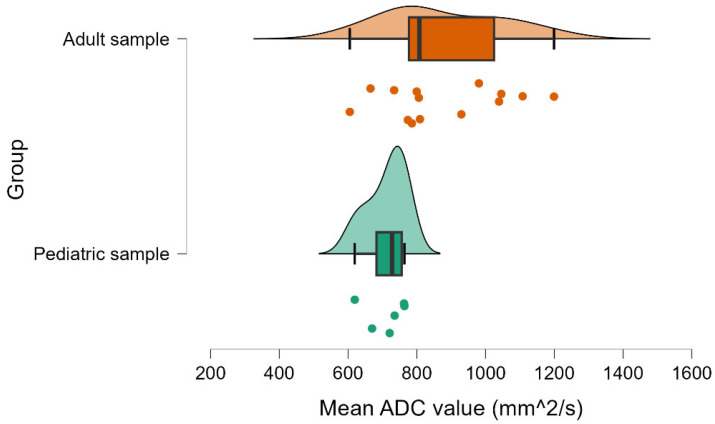
Group differences regarding mean ADC values.

**Figure 5:**
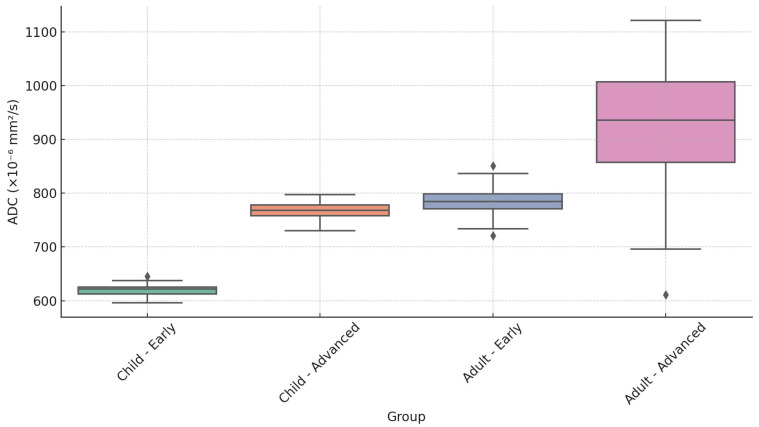
Distribution of apparent diffusion coefficient values by age and tumor stage in pediatric and adult cohorts. ◆ indicates outliers beyond 1.5× the interquartile range (IQR).

We found a statistically significant difference between the mean ADC values of pediatric patients and adult patients, t (17.44) = −3.15, *p* = 0.006, with the mean ADC values of pediatric patients (M = 712.22, standard deviation [SD] = 57.03) being lower, on average, than the mean ADC values of adult patients (M = 877.34, SD = 175.25; Figure 4). The effect size for the difference between the means of the pediatric and adult patients was large (d = −1.26, 95% CI: −2.29 to −0.20). A detailed breakdown of ADC values by age group and T-stage, initially summarized in Table 1, is visually represented in the boxplot shown in Figure 5.

## 4. Discussion

We evaluated the pre-treatment baseline ADC values between pediatric and adult populations in our retrospective MRI investigation. The mean ADC extracted from the initial pre-treatment DWI-MRI in children/adolescents was 712.22 × 10^−6^ mm^2^/s, compared to adults in whom the mean ADC was 877.34 × 10^−6^ (0.874 × 10^−3^) mm^2^/s. Our main finding was that children and adolescents with NPC had significantly lower initial ADC values compared with adults.

Our study highlights important differences in the diffusion characteristics of nasopharyngeal carcinoma (NPC) between pediatric and adult patients. Specifically, we found that mean ADC values were significantly lower in pediatric patients compared to adults, as demonstrated by independent samples *t*-test. This suggests that age-related biological differences may influence the diffusion properties of NPC tumors, potentially reflecting underlying differences in tumor cellularity, extracellular matrix composition, or tissue microstructure.

Pediatric NPC represents less than 1% of all childhood cancers [2]. The peak of incidence is between 10 and 20 years, with a median age of 13 years at diagnosis [5]. This age peak seems to be in concordance with our cohort, given that the mean age of the included children/adolescents was 13.1 ± 4.1 years at pre-treatment MRI. Multi-sequence MRI may be the most powerful modality in the field of pediatric or adult oncology for staging, allowing us to make assumptions about the treatment response and to identify disease relapse, particularly in HNC cancers, including NPC. Previous studies have demonstrated that the initial ADC values of NPC represent a viable prognostic marker for treatment [32,33]. Therapeutic response monitoring is another possibility by comparing the changes in free water diffusivity within the tumor after treatment [34]. A study by Zhang et al. showed that NPC patients with lower ADC values of their primary nasopharyngeal tumors measured on pre-treatment DWI-MRI scans were better responders to neoadjuvant chemotherapy [35]. A similar pattern of results was observed by Yu et al., who reviewed NPC patients with the help of intravoxel incoherent motion (IVIM) DWI-MRI scans [36]. They reported that patients who were effectively treated with induction chemotherapy and concurrent chemo-RT had lower ADC values on their pre-treatment DWI-MRI scans.

Importantly, to control for the potential confounding effect of tumor T-stage, we performed a two-way ANOVA including age group and T-stage (early vs. advanced). This analysis confirmed that age group remained a statistically significant factor influencing ADC values (F = 5.03, *p* = 0.040), whereas T-stage alone did not reach statistical significance (F = 2.89, *p* = 0.109), and no significant interaction was observed (F = 0.74, *p* = 0.403). Therefore, it appears that tumor stage has little bearing on the reported differences in ADC between children and adults.

Several studies have reported that the baseline ADC values of nasopharyngeal tumors may be a prognostic indicator for treatment [37,38,39]. Our current study demonstrates that children/adolescents with NPC had significantly lower ADC values as compared with adults, and these findings may bring a new perspective for the prognosis for NPC. The results may provide evidence that children/adolescents have a better prognosis than adults. In general, the disease prognosis is generally good, with 5-year survival higher than 80% [40]. The main cause of treatment failure in pediatric NPC is distant metastasis [41].

Additionally, descriptive statistics indicated a trend toward lower ADC values in early-stage tumors compared to advanced-stage tumors, particularly among pediatric patients. These results should be interpreted cautiously, nevertheless, because there was only one early-stage pediatric case (n = 1). In adults, ADC values were generally higher, especially in advanced-stage disease. These patterns are consistent with earlier research showing that advanced NPC may have greater ADC values as a result of less dense tumor cellularity or increased necrosis.

Our mean ADC value for children/adolescents was 712.22 × 10^−6^ mm^2^/s, and for adults, the mean ADC value was 877.34 × 10^−6^ mm^2^/s. Previous work divided ADC values into two categories: low ADC and high ADC groups [37]. A study conducted by Zhang et al. reported that an ADC value lower than 747 × 10^−6^ mm^2^/s was considered for the low ADC group, and they found that the low ADC group had better 3-year local relapse-free survival (LRFS) than the high ADC group [25]. Our results demonstrate that only children/adolescents had values corresponding to such a low ADC group. This may suggest that children/adolescents have a good prognostic perspective for NPC compared with adults.

Our findings contribute to the growing body of evidence suggesting that both patient age and tumor stage can influence diffusion imaging metrics, but that age-related differences may be particularly pronounced in NPC. Future studies with larger cohorts are warranted to further delineate these relationships and to explore their potential clinical implications for imaging-based tumor characterization and treatment planning.

The ADC values can provide useful information to predict the outcome and to identify high-risk patients qualified for more aggressive therapy. The type of treatment depends on the tumor stage, according to the 8th Edition of the American Joint Committee on Cancer Staging System [42]. Surgery is an exceptional option for the treatment of NPC [43]. For stage I or II (N0), children and adolescents receive exclusive RT, leading to a 98% 10-year OS rate [44]. On the other hand, advanced stages of pediatric NPC treated historically by RT alone have demonstrated poor prognosis, with a 4-year disease-free survival of about 40% due to metastatic relapses [41]. The effective impact of chemotherapy in addition to RT has been clearly shown in adult populations [45]. Several retrospective studies have reported a better survival rate after combined treatment in pediatric series [37,38,39,46]. Combined treatment has become the standard in the pediatric population considering the survival improvement reported in more recent prospective studies for stages II (N1), III, and Iva [34,46,47]. Metastatic patients (IVb) are treated with a multimodal strategy with initial chemotherapy regimens, loco-regional RT, whenever possible, focal treatment of metastases, and maintenance therapy [46].

To our knowledge, this is the first study comparing the pre-treatment ADC values between children and adults. According to Zhang et al., integrating pre-treatment ADC values with the existing T classification provided better prognostic accuracy for local failure than the T classification alone in patients with NPC [25]. The mechanism linking a high pre-treatment ADC value to local failure may be associated with the tumor’s radiosensitivity. Several biological factors, including hypoxia, inflammation, cell density, and cell membrane integrity, can influence water diffusion in tissues, thereby affecting the ADC value [48]. These factors may also impact the tumor’s radiosensitivity. For instance, tumor hypoxia promotes inflammation [49], which is associated with elevated ADC values due to an increase in interstitial water content [50]. Additionally, hypoxia is a well-established and characteristic feature linked to reduced radiosensitivity [51]. Tumors with increased cell density (i.e., lower ADC values) tend to have a greater number of viable proliferative cells, potentially resulting in a more effective response to RT [52].

Our research has the potential to be a valuable approach in the early identification of children with low ADC who would benefit from a personalized treatment strategy. Patients with high pre-treatment ADC values may benefit from initial aggressive treatment approaches, including the incorporation of molecularly targeted therapies or radiosensitizers like sodium glycididazole [53]. However, the effectiveness of these strategies should be explored in future research. In our view, this study’s results represent a relevant step towards using diffusion MRI as part of the imaging protocol in the evaluation of nasopharyngeal tumors. Specifically, DWI-MRI allows for non-invasive in vivo evaluation of the intrinsic biological properties of tissues and has demonstrated its utility in differential diagnosis, staging, therapy response monitoring, prognostic assessment, and early detection of recurrence in various malignancies [54,55,56].

The study’s strengths rely on a high intraclass correlation coefficient (ICC = 0.98) indicates that the research exhibits excellent accuracy in ADC value estimation, which lessens the possibility of researcher bias and increases the data’ reliability. Appropriate statistical tests were applied for this set of data, and all the results show statistical significance. The research’s strong distinction between adolescent and adult subjects enables careful examinations of variations in ADC levels within both groups. The study was conducted at three healthcare facilities; therefore, the results might be more broadly applicable.

The major limitations of this study are the small number of patients as well as the lack of follow-up MRI examinations. A larger sample size would increase statistical power and offer more reliable findings, and the narrow age range for adults might not accurately reflect ADC differences of adults with NPC. Specifically, in this study, we have not analyzed post-treatment ADC values from additional MRI investigations for both children/adolescents and adults to confirm our hypothesis regarding prognostic perspectives of ADC values (and especially regarding the difference in ADC values between pediatric and adult patients). It is challenging to evaluate how ADC values vary over time or in relation to treatment. However, considering this rare pathology in Europe, especially among children, and considering that the data were collected from three medical centers, the data should still be valuable to provide evidence of significant differences between children/adolescents and adult patients. Another limitation of the study is the variety of parameters and MRI scanners that were used to acquire the imaging data. Nevertheless, because the ADC parameter is a computed parameter using a standard computation mode, those acquisition parameters are not considered susceptible to strong influences for the analysis of this study.

## 5. Conclusions

This study compared the baseline ADC values of pediatric and adult NPC patients from pre-treatment MRI investigations. Our results showed significant differences between groups, demonstrating lower ADC values in pediatric patients compared to the adult population. These findings may help identify pediatric NPC patients with markedly low pre-treatment ADC values, which could indicate a distinct tumor microenvironment compared to adults. While lower ADC values in adult NPC have often been associated with favorable treatment response, pediatric NPC is known to exhibit more aggressive tumor biology, yet paradoxically better treatment outcomes, consistent with previous reports. This highlights the need for age-specific interpretation of ADC metrics and supports further research into their biological underpinnings in pediatric cases.

Importantly, our analysis demonstrated that the observed differences in ADC values between age groups were independent of tumor T-stage, reinforcing the primary influence of age on diffusion characteristics. Additionally, descriptive statistics indicated a trend toward lower ADC values in early-stage tumors compared to advanced-stage tumors, particularly among pediatric patients.

Overall, this study represents an important first step toward the use of diffusion MRI for evaluating head and neck malignancies. The findings may help with better diagnosis and treatment planning for individuals with NPC and highlight the importance of taking age-related variations in ADC values into account when interpreting DWI-MRI data.

## Figures and Tables

**Table 1:** Summary of apparent diffusion coefficient (ADC) values (×10^−6^ mm^2^/s) by age and tumor stage.

Age Group	Tumor Stage	Minimum	Median	Maximum
Child	Early (T1–T2)	590	620	650
Child	Advanced (T3–T4)	730	770	810
Adult	Early (T1–T2)	665	785	806
Adult	Advanced (T3–T4)	605	930	1200

**Table 2:** Clinical and pathological characteristics of children and adults with NPC.

Characteristic	No. of Children n = 6 (%)	No. of Adults n = 14 (%)
Age (years)		
Median	16	58
Range	12–17	34–70
Gender		
Male	3 (50)	10 (71.4)
Female	3 (50)	4 (28.6)
Pathologic Type ^1^		
keratinizing squamous carcinoma	0 (0.0)	0 (0.0)
differentiated/undifferentiated non-keratinizing carcinoma	6 (100.0)	14 (100.0)
basaloid squamous carcinoma	0 (0.0)	0 (0.0)
T Category ^2^		
T1	1 (16.7)	0 (0.0)
T2	1 (16.7)	4 (28.6)
T3	1 (16.7)	4 (28.6)
T4	3 (50.0)	6 (42.9)
N Category ^2^		
N0	0 (0.0)	3 (21.4)
N1	2 (33.3)	0 (0.0)
N2	3 (50.0)	6 (42.9)
N3	1 (16.7)	5 (35.7)
Stage ^2^		
I	0 (0.0)	0 (0.0)
II	1 (16.7)	1 (7.1)
III	0 (0.0)	3 (21.4)
IV A/B	5 (83.3)	10 (71.4)

Clinical features of the 20 patients with NPC. Abbreviations: NPC—nasopharyngeal carcinoma; n—sample size. ^1^ Pathologic type, according to the 2017 World Health Organization classification of tumors [30], and ^2^ according to the 8th UICC/AJCC staging system [31].

## Data Availability

The data presented in this study are available in this article.

## Abbreviations

The following abbreviations are used in this manuscript:

HNC	Head and neck
RMS	Rhabdomyosarcomas
NPC	Nasopharyngeal carcinoma
SCC	Squamous cell carcinoma
WHO	World Health Organization
EBV	Epstein–Barr virus
RT	Radiotherapy
OS	Overall survival
MRI	Magnetic resonance imaging
CT	Computed tomography
18 FDG PET-CT	18F-fluoro-deoxy-glucose positron emission tomography
DWI	Diffusion-weighted imaging
ADC	Apparent diffusion coefficient
AFNI	Analysis of Functional NeuroImages
ROI	Region of interest
ICC	Intraclass correlation coefficient
CI	Confidence intervals
PET	Positron emission tomography

## References

[1] Grønhøj C., Hjalgrim L., Jakobsen K.K., Charabi B., Mirian C., Laier G.H., Kiss K., Rechnitzer C., Friborg J., Von Buchwald C. (2018). Incidence of Head and Neck Cancer in Children: A Danish Nationwide Study from 1978 to 2014. Pediatr. Blood Cancer.

[2] Qaisi M., Eid I. (2016). Pediatric Head and Neck Malignancies. Oral. Maxillofac. Surg. Clin. N. Am..

[3] Lopez J., Tufaro A.P. (2024). Head and Neck Malignancies in Children. Oral. Maxillofac. Surg. Clin. N. Am..

[4] Ben-Ami T. (2024). Nasopharyngeal Carcinoma in Children, Current Treatment Approach. J. Pediatr. Hematol. Oncol..

[5] Claude L., Jouglar E., Duverge L., Orbach D. (2019). Update in Pediatric Nasopharyngeal Undifferentiated Carcinoma. Br. J. Radiol..

[6] Hasnaoui M., Lahmar R., Ben Mabrouk A., Masmoudi M., Mighri K., Driss N. (2020). Predictive Epidemiological and Clinical Factors of Nasopharyngeal Carcinoma Diagnosis: Adult versus Pediatric Population. Int. J. Pediatr. Otorhinolaryngol..

[7] Chang E.T., Ye W., Zeng Y.-X., Adami H.-O. (2021). The Evolving Epidemiology of Nasopharyngeal Carcinoma. Cancer Epidemiol. Biomark. Prev..

[8] Sultan I., Casanova M., Ferrari A., Rihani R., Rodriguez-Galindo C. (2010). Differential Features of Nasopharyngeal Carcinoma in Children and Adults: A SEER Study. Pediatr. Blood Cancer.

[9] Chen Y.-P., Chan A.T.C., Le Q.-T., Blanchard P., Sun Y., Ma J. (2019). Nasopharyngeal Carcinoma. Lancet.

[10] Ayan I., Kaytan E., Ayan N. (2003). Childhood Nasopharyngeal Carcinoma: From Biology to Treatment. Lancet Oncol..

[11] Ben-Ami T., Kontny U., Surun A., Brecht I.B., Almaraz R.L., Dragomir M., Pourtsidis A., Casanova M., Fresneau B., Bisogno G. (2021). Nasopharyngeal Carcinoma in Children and Adolescents: The EXPeRT/PARTNER Diagnostic and Therapeutic Recommendations. Pediatr. Blood Cancer.

[12] Sun Y., Li W.-F., Chen N.-Y., Zhang N., Hu G.-Q., Xie F.-Y., Sun Y., Chen X.-Z., Li J.-G., Zhu X.-D. (2016). Induction Chemotherapy plus Concurrent Chemoradiotherapy versus Concurrent Chemoradiotherapy Alone in Locoregionally Advanced Nasopharyngeal Carcinoma: A Phase 3, Multicentre, Randomised Controlled Trial. Lancet Oncol..

[13] Rodriguez-Galindo C., Krailo M.D., Krasin M.J., Huang L., McCarville M.B., Hicks J., Pashankar F., Pappo A.S. (2019). Treatment of Childhood Nasopharyngeal Carcinoma With Induction Chemotherapy and Concurrent Chemoradiotherapy: Results of the Children’s Oncology Group ARAR0331 Study. J. Clin. Oncol..

[14] Chen J., Luo J., He X., Zhu C. (2020). Evaluation of Contrast-Enhanced Computed Tomography (CT) and Magnetic Resonance Imaging (MRI) in the Detection of Retropharyngeal Lymph Node Metastases in Nasopharyngeal Carcinoma Patients. Cancer Manag. Res..

[15] Quartuccio N., Pulizzi S., Modica D.M., Nicolosi S., D’Oppido D., Moreci A.M., Ialuna S. (2024). Head-to-Head Comparison of [18F]FDG PET Imaging and MRI for the Detection of Recurrence or Residual Tumor in Patients with Nasopharyngeal Carcinoma: A Meta-Analysis. Cancers.

[16] Xie C., Vardhanabhuti V. (2022). PET/CT. PET Clin..

[17] Mui A.W.L., Lee A.W.M., Lee V.H.F., Ng W.T., Vardhanabhuti V., Man S.S.Y., Chua D.T.T., Law S.C.K., Guan X.Y. (2021). Prognostic and Therapeutic Evaluation of Nasopharyngeal Carcinoma by Dynamic Contrast-Enhanced (DCE), Diffusion-Weighted (DW) Magnetic Resonance Imaging (MRI) and Magnetic Resonance Spectroscopy (MRS). Magn. Reson. Imaging.

[18] King A.D. (2022). MR Imaging of Nasopharyngeal Carcinoma. Magn. Reson. Imaging Clin. N. Am..

[19] Orman G., Tran B.H., Desai N., Meoded A., Kralik S., Smith V., Hicks J., Kirsch C., Huisman T.A.G.M. (2021). Neuroimaging Characteristics of Nasopharyngeal Carcinoma in Children. J. Neuroimaging.

[20] Sheppard S.C., Giger R., Bojaxhiu B., Sachpekidis C., Dammann F., Dettmer M.S., Arnold A., Wartenberg J., Nisa L. (2021). Multimodal Imaging With Positron Emission Tomography/Computed Tomography and Magnetic Resonance Imaging to Detect Extracapsular Extension in Head and Neck Cancer. Laryngoscope.

[21] Mohandas A., Marcus C., Kang H., Truong M.-T., Subramaniam R.M. (2014). FDG PET/CT in the Management of Nasopharyngeal Carcinoma. AJR Am. J. Roentgenol..

[22] Guo R., Mao Y.-P., Tang L.-L., Chen L., Sun Y., Ma J. (2019). The Evolution of Nasopharyngeal Carcinoma Staging. Br. J. Radiol..

[23] Abdel Razek A.A.K., Gaballa G., Elhawarey G., Megahed A.S., Hafez M., Nada N. (2009). Characterization of Pediatric Head and Neck Masses with Diffusion-Weighted MR Imaging. Eur. Radiol..

[24] King A.D., Vlantis A.C., Yuen T.W.C., Law B.K.H., Bhatia K.S., Zee B.C.Y., Woo J.K.S., Chan A.T.C., Chan K.C.A., Ahuja A.T. (2015). Detection of Nasopharyngeal Carcinoma by MR Imaging: Diagnostic Accuracy of MRI Compared with Endoscopy and Endoscopic Biopsy Based on Long-Term Follow-Up. AJNR Am. J. Neuroradiol..

[25] Zhang Y., Liu X., Zhang Y., Li W.-F., Chen L., Mao Y.-P., Shen J.-X., Zhang F., Peng H., Liu Q. (2015). Prognostic Value of the Primary Lesion Apparent Diffusion Coefficient (ADC) in Nasopharyngeal Carcinoma: A Retrospective Study of 541 Cases. Sci. Rep..

[26] Lai V., Li X., Lee V.H.F., Lam K.O., Chan Q., Khong P.L. (2013). Intravoxel Incoherent Motion MR Imaging: Comparison of Diffusion and Perfusion Characteristics between Nasopharyngeal Carcinoma and Post-Chemoradiation Fibrosis. Eur. Radiol..

[27] https://afni.nimh.nih.gov.

[28] (2018). IBM SPSS Statistics for Windows SPSS.

[29] (2024). JASP.

[30] Stelow E.B., Wenig B.M. (2017). Update from The 4th Edition of the World Health Organization Classification of Head and Neck Tumours: Nasopharynx. Head Neck Pathol..

[31] Tang L.-L., Chen Y.-P., Mao Y.-P., Wang Z.-X., Guo R., Chen L., Tian L., Lin A.-H., Li L., Sun Y. (2017). Validation of the 8th Edition of the UICC/AJCC Staging System for Nasopharyngeal Carcinoma From Endemic Areas in the Intensity-Modulated Radiotherapy Era. J. Natl. Compr. Cancer Netw..

[32] Law B.K.H., King A.D., Bhatia K.S., Ahuja A.T., Kam M.K.M., Ma B.B., Ai Q.Y., Mo F.K.F., Yuan J., Yeung D.K.W. (2016). Diffusion-Weighted Imaging of Nasopharyngeal Carcinoma: Can Pretreatment DWI Predict Local Failure Based on Long-Term Outcome?. AJNR Am. J. Neuroradiol..

[33] Das A., Bhalla A.S., Sharma R., Kumar A., Thakar A., Vishnubhatla S.M., Sharma M.C., Sharma S.C. (2017). Can Diffusion Weighted Imaging Aid in Differentiating Benign from Malignant Sinonasal Masses?: A Useful Adjunct. Pol. J. Radiol..

[34] Chen Y., Liu X., Zheng D., Xu L., Hong L., Xu Y., Pan J. (2014). Diffusion-Weighted Magnetic Resonance Imaging for Early Response Assessment of Chemoradiotherapy in Patients with Nasopharyngeal Carcinoma. Magn. Reson. Imaging.

[35] Zhang G.-Y., Wang Y.-J., Liu J.-P., Zhou X.-H., Xu Z.-F., Chen X.-P., Xu T., Wei W.-H., Zhang Y., Huang Y. (2015). Pretreatment Diffusion-Weighted MRI Can Predict the Response to Neoadjuvant Chemotherapy in Patients with Nasopharyngeal Carcinoma. BioMed Res. Int..

[36] Yu X.-P., Hou J., Li F.-P., Wang H., Hu P.-S., Bi F., Wang W. (2016). Intravoxel Incoherent Motion Diffusion Weighted Magnetic Resonance Imaging for Differentiation Between Nasopharyngeal Carcinoma and Lymphoma at the Primary Site. J. Comput. Assist. Tomogr..

[37] Huang T., Lu N., Lian S., Li H., Yin S., Geng Z., Xie C. (2019). The Primary Lesion Apparent Diffusion Coefficient Is a Prognostic Factor for Locoregionally Advanced Nasopharyngeal Carcinoma: A Retrospective Study. BMC Cancer.

[38] Yan D.-F., Zhang W.-B., Ke S.-B., Zhao F., Yan S.-X., Wang Q.-D., Teng L.-S. (2017). The Prognostic Value of Pretreatment Tumor Apparent Diffusion Coefficient Values in Nasopharyngeal Carcinoma. BMC Cancer.

[39] Liu L.-T., Guo S.-S., Li H., Lin C., Sun R., Chen Q.-Y., Liang Y.-J., Tang Q.-N., Sun X.-S., Tang L.-Q. (2021). Percent Change in Apparent Diffusion Coefficient and Plasma EBV DNA after Induction Chemotherapy Identifies Distinct Prognostic Response Phenotypes in Advanced Nasopharyngeal Carcinoma. BMC Cancer.

[40] Sinha S., Winters R., Gajra A. (2025). Nasopharyngeal Cancer. StatPearls.

[41] Liu W., Tang Y., Gao L., Huang X., Luo J., Zhang S., Wang K., Qu Y., Xiao J., Xu G. (2014). Nasopharyngeal Carcinoma in Children and Adolescents—A Single Institution Experience of 158 Patients. Radiat. Oncol..

[42] Amin M.B., Greene F.L., Edge S.B., Compton C.C., Gershenwald J.E., Brookland R.K., Meyer L., Gress D.M., Byrd D.R., Winchester D.P. (2017). The Eighth Edition AJCC Cancer Staging Manual: Continuing to Build a Bridge from a Population-based to a More “Personalized” Approach to Cancer Staging. CA Cancer J. Clin..

[43] Lee A.W.M., Ng W.T., Chan J.Y.W., Corry J., Mäkitie A., Mendenhall W.M., Rinaldo A., Rodrigo J.P., Saba N.F., Strojan P. (2019). Management of Locally Recurrent Nasopharyngeal Carcinoma. Cancer Treat. Rev..

[44] Karray H., Ayadi W., Fki L., Hammami A., Daoud J., Drira M.M., Frikha M., Jlidi R., Middeldorp J.M. (2005). Comparison of Three Different Serological Techniques for Primary Diagnosis and Monitoring of Nasopharyngeal Carcinoma in Two Age Groups from Tunisia. J. Med. Virol..

[45] Baujat B., Audry H., Bourhis J., Chan A.T.C., Onat H., Chua D.T.T., Kwong D.L.W., al-Sarraf M., Chi K.-H., Hareyama M. (2006). Chemotherapy in Locally Advanced Nasopharyngeal Carcinoma: An Individual Patient Data Meta-Analysis of Eight Randomized Trials and 1753 Patients. Int. J. Radiat. Oncol. Biol. Phys..

[46] Casanova M., Bisogno G., Gandola L., Cecchetto G., Di Cataldo A., Basso E., Indolfi P., D’Angelo P., Favini F., Collini P. (2012). A Prospective Protocol for Nasopharyngeal Carcinoma in Children and Adolescents: The Italian Rare Tumors in Pediatric Age (TREP) Project. Cancer.

[47] Rodriguez-Galindo C., Wofford M., Castleberry R.P., Swanson G.P., London W.B., Fontanesi J., Pappo A.S., Douglass E.C. (2005). Preradiation Chemotherapy with Methotrexate, Cisplatin, 5-fluorouracil, and Leucovorin for Pediatric Nasopharyngeal Carcinoma: Results of Pediatric Oncology Group (Now Children’s Oncology Group) Study 9486. Cancer.

[48] Hatakenaka M., Nakamura K., Yabuuchi H., Shioyama Y., Matsuo Y., Ohnishi K., Sunami S., Kamitani T., Setoguchi T., Yoshiura T. (2011). Pretreatment Apparent Diffusion Coefficient of the Primary Lesion Correlates With Local Failure in Head-and-Neck Cancer Treated with Chemoradiotherapy or Radiotherapy. Int. J. Radiat. Oncol. Biol. Phys..

[49] Eltzschig H.K., Carmeliet P. (2011). Hypoxia and Inflammation. N. Engl. J. Med..

[50] Murakami R., Sugahara T., Nakamura H., Hirai T., Kitajima M., Hayashida Y., Baba Y., Oya N., Kuratsu J., Yamashita Y. (2007). Malignant Supratentorial Astrocytoma Treated with Postoperative Radiation Therapy: Prognostic Value of Pretreatment Quantitative Diffusion-Weighted MR Imaging. Radiology.

[51] Brizel D.M., Sibley G.S., Prosnitz L.R., Scher R.L., Dewhirst M.W. (1997). Tumor Hypoxia Adversely Affects the Prognosis of Carcinoma of the Head and Neck. Int. J. Radiat. Oncol. Biol. Phys..

[52] Hatakenaka M., Shioyama Y., Nakamura K., Yabuuchi H., Matsuo Y., Sunami S., Kamitani T., Yoshiura T., Nakashima T., Nishikawa K. (2011). Apparent Diffusion Coefficient Calculated with Relatively High B-Values Correlates with Local Failure of Head and Neck Squamous Cell Carcinoma Treated with Radiotherapy. AJNR Am. J. Neuroradiol..

[53] Sung F.L., Pang R.T.K., Ma B.B.Y., Lee M.M.L., Chow S.M., Poon T.C.W., Chan A.T.C. (2006). Pharmacoproteomics Study of Cetuximab in Nasopharyngeal Carcinoma. J. Proteome Res..

[54] Vandecaveye V., De Keyzer F., Vander Poorten V., Dirix P., Verbeken E., Nuyts S., Hermans R. (2009). Head and Neck Squamous Cell Carcinoma: Value of Diffusion-Weighted MR Imaging for Nodal Staging. Radiology.

[55] Wu L.-M., Chen X.-X., Li Y.-L., Hua J., Chen J., Hu J., Xu J.-R. (2014). On the Utility of Quantitative Diffusion-Weighted MR Imaging as a Tool in Differentiation between Malignant and Benign Thyroid Nodules. Acad. Radiol..

[56] Thoeny H.C., De Keyzer F., King A.D. (2012). Diffusion-Weighted MR Imaging in the Head and Neck. Radiology.

